# Supplementary material for: The evolutionary history of bears is characterized by gene flow across species
Source: Sci Rep. 2017 Apr 19;7:46487. doi: 10.1038/srep46487 (PMC5395953; doi:10.1038/srep46487)
Supplement: Supplementary Information [file srep46487-s1.pdf]

Supplementary Information

The evolutionary history of bears is characterized by gene flow across species

Vikas Kumar, Fritjof Lammers, Tobias Bidon, Markus Pfenninger, Lydia Kolter, Maria A. Nilsson, Axel Janke *

*correspondence to: Axel.Janke@senckenberg.de

Supplementary Figures 1-22

Supplementary Tables 1-7

Supplementary Methods and References

Supplementary Figures

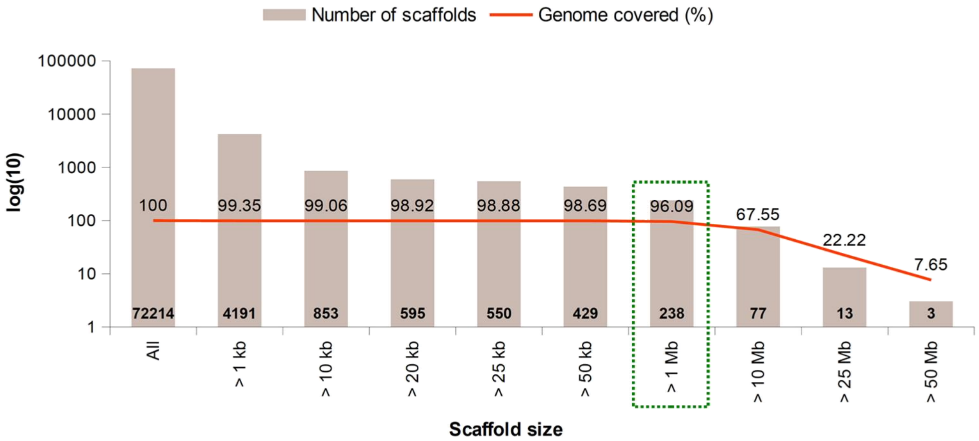

Supplementary Fig. 1. Distribution of scaffolds lengths and percentage of polar bear reference genome coverage in bins of different scaffold sizes. The figure shows the percentage (number above the red line) of genome coverage by the scaffolds. Bold face numbers show the number of scaffolds in the respective bin. Scaffolds >1 Mb cover >96% (highlighted by the dashed green box) of the polar bear genome and were used as a reference for mapping the reads of the other bear genomes.

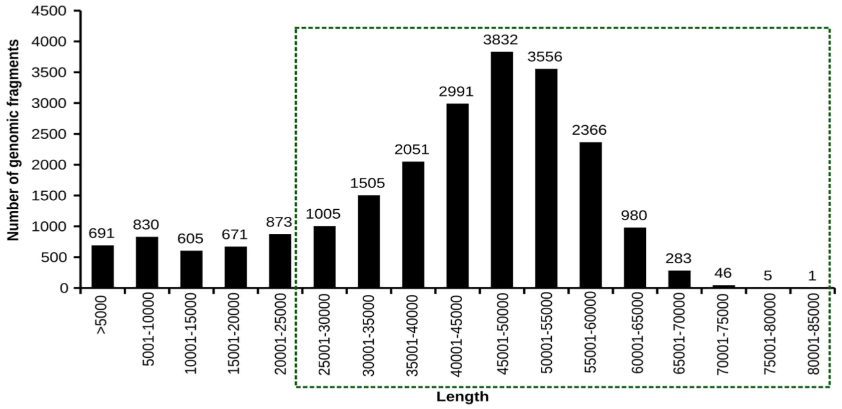

Supplementary Fig. 2. Length distribution of genomic fragments after the removal of Ns, gaps and repeat elements. The highlighted region with the dashed green box shows the GFs with a length greater than 25,000 bp (mean sequence length of 46,685 bp, standard deviation of 9,490 bp) that were used for further analyses. The number above each bar represents the total number of fragments in each bin. The total length of the 18,621 GF> 25 kb (dotted square) is 869,313,834 bp.

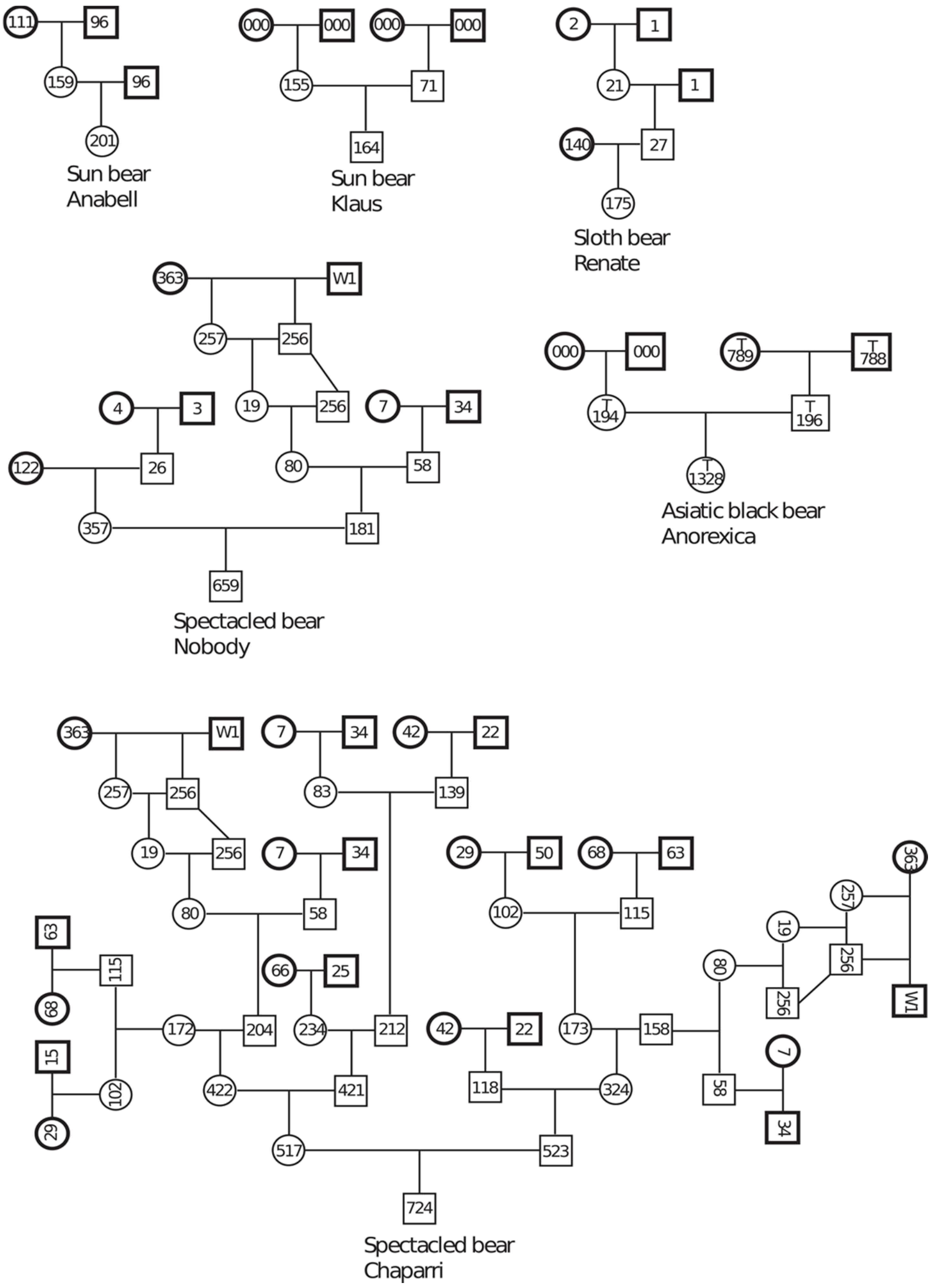

Supplementary Fig. 3. Pedigrees of the captive individuals used in this study. Circle indicates female, square male. Thick-lined symbols represent individuals captured in the wild, the numbers refer to the studbook number of the International (Chaparri, Nobody) or European (others) Studbooks [1–4]. The names are the individual's house-name. "000" indicates that for these wild individuals no studbook number or name is recorded, but they were representative of their species for a captive breeding program. The breeding success was low in the 60s and 70s and therefore

none of the captive bred animals could be linked to these “000” making these most likely wild born individuals, which are unlikely hybrids when they were included in a breeding program. Thus, none of the studied individuals or their ancestors were species hybrids. The inbreeding coefficient for Chaparri is only 0.0053, despite the apparent inbreeding of the ancestors.

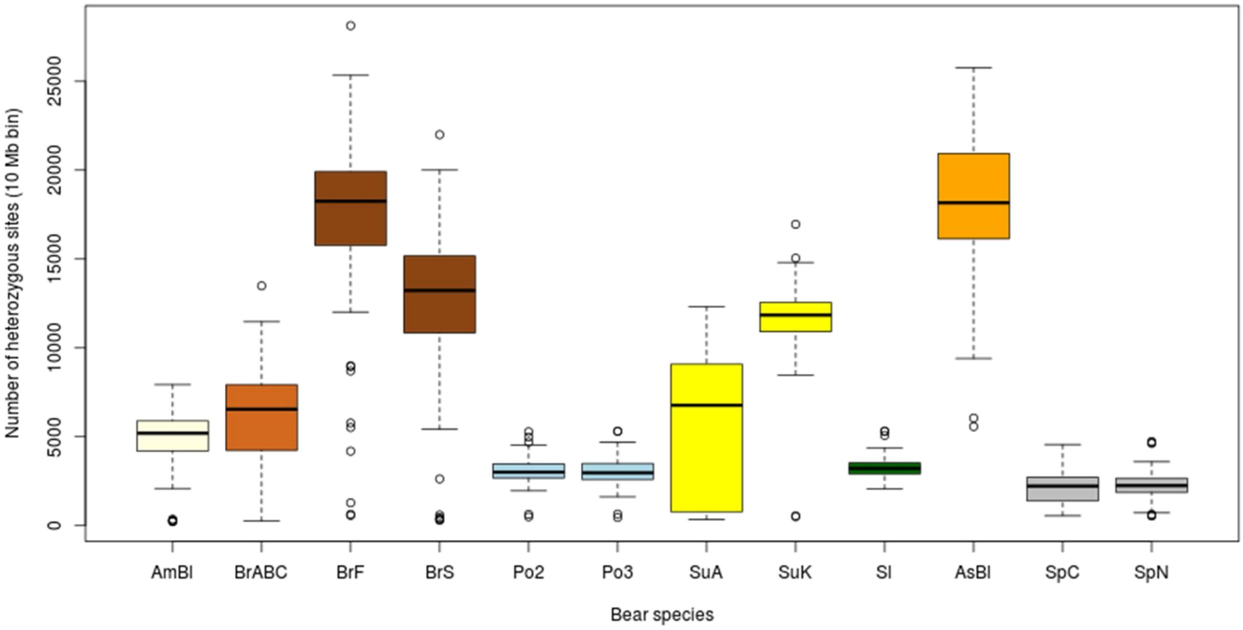

Supplementary Fig. 4. Box plot showing the number of heterozygous sites in 10 Mb bins for all bear genomes. The x-axis shows the bear species and the y-axis depicts the absolute number of heterozygous sites per 10 Mb fragment. AmBl: American black bear, BrABC: Brown bear ABC, BrF: Brown bear -Finland, BrS: Brown bear -Sweden, Po2: Polar bear -2, Po3: Polar bear 3, SuA: Sun bear- Anabell, SuK: Sun bear -Klaus, Sl: Sloth bear, AsBl: Asiatic black bear, SpC: Spectacled bear- Chappari and SpN: Spectacled bear – Nobody.

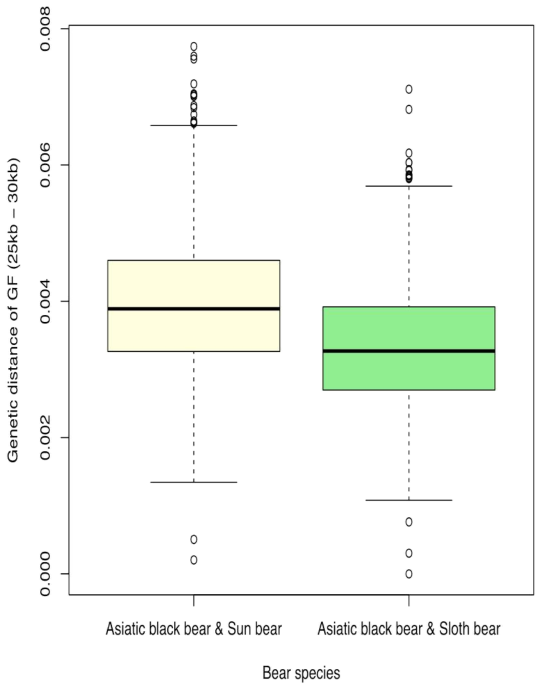

Supplementary Fig. 5. Box plot of pair wise distances between the Asiatic black bear to sun bear and sloth bear for GFs 25-30 kb. Each box represents the interquartile range with outliers. The average number of substitutions is about 104 bp per GF, with few outliers, indicating sufficient phylogenetic signal among bears for each fragment to distinguish alternative trees.

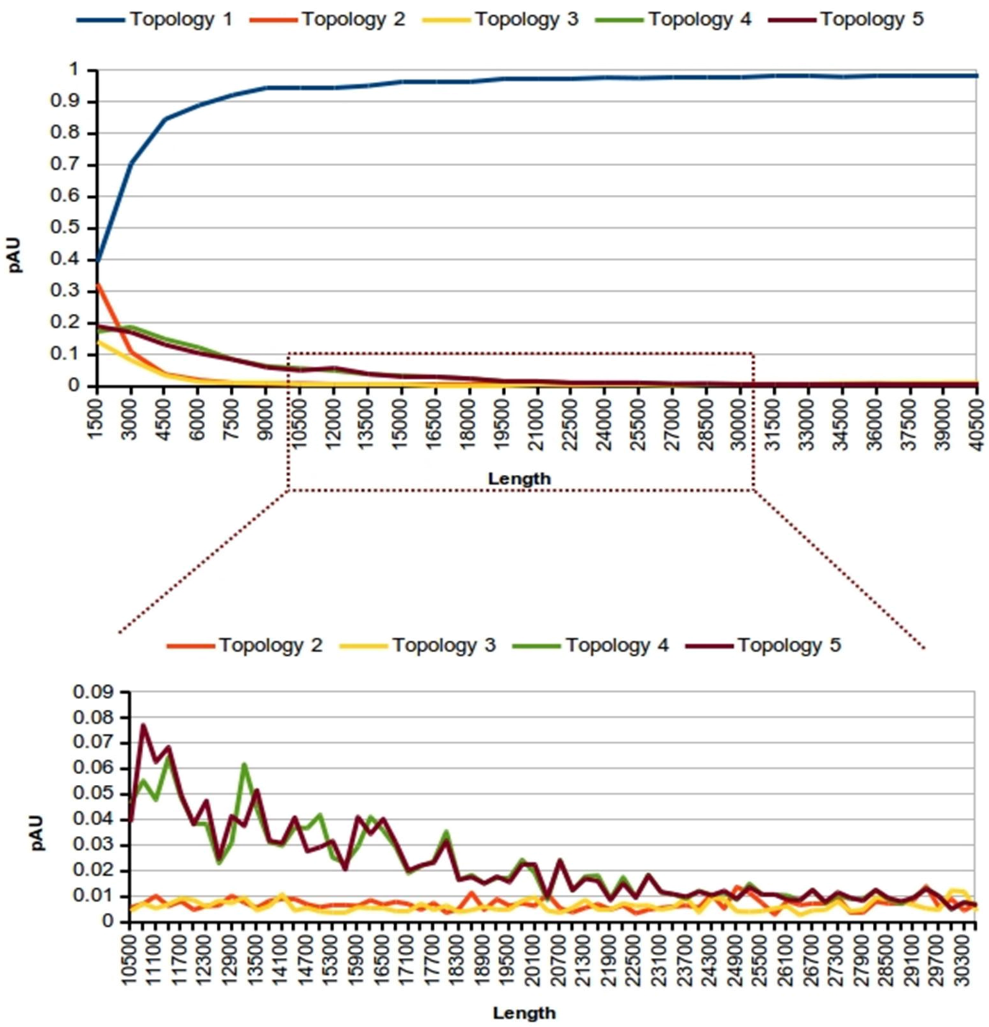

Supplementary Fig. 6. Computer simulation to evaluate the length of GFs needed to significantly support or reject a topology. Five different topologies were tested. Based on the parameters of the species tree (Topology 1, Fig 2A), data sets with increasing lengths were simulated and the AU values calculated for the five topologies. Topology 2 (mtDNA tree), which is the most deviating, and Topology 3 (American plus and Brown bear) is rejected rather quickly, while the less deviant topologies “4” (Asiatic black bear and sloth bear as sister group and “5” (Asiatic black bear and sun bear as sister group) require longer sequences to be rejected. pAU – AU probability value.

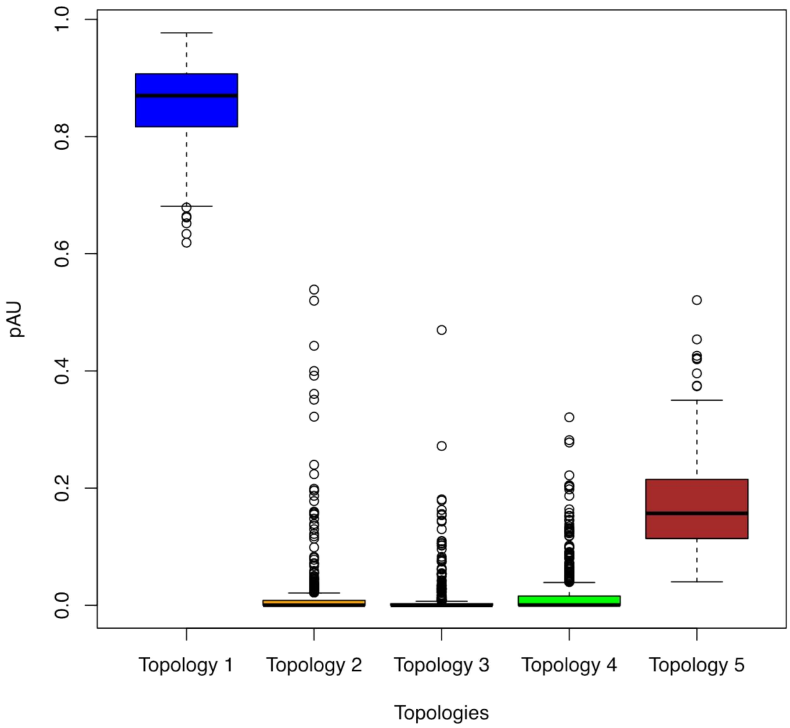

Supplementary Fig. 7. Maximum likelihood test statistics (AU) of five topologies (see Supplementary Fig. 6). The analysis included 500 random GF data sets that reconstruct coalescent species tree (Fig. 2A) as the best ML tree. Topology 1 received on average AU probabilities >0.85, Topology 2, 3 and 4 are significantly rejected by nearly all data sets and Topology 5 cannot be significantly rejected. Thus, the majority of GFs that support a particular tree do so in nearly all cases with significant support. Topologies are given in Supplementary Fig. 6 legend. pAU – AU probability value.

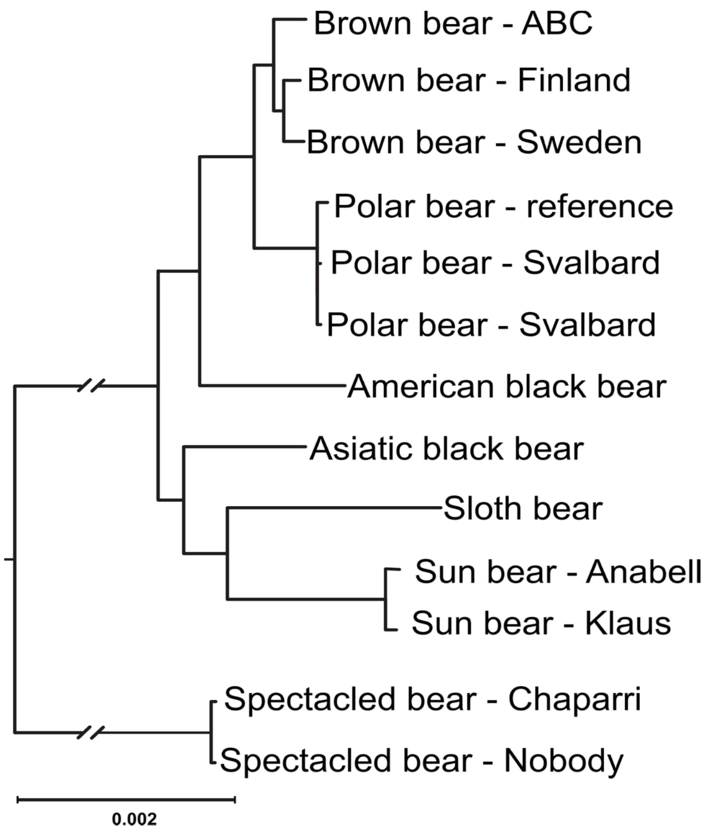

Supplementary Fig. 8. Phylogenetic relationship among the bears using GFs. The coalescent species tree of 18,621 individual GFs >25 kb together with names or geographical origin of the individual. All branches received 100% bootstrap support. The position of the root in the tree, as well as the depicted branch lengths were calculated from 10 Mb of GF sequences. The scale bar indicates 0.002 substitutions per site.

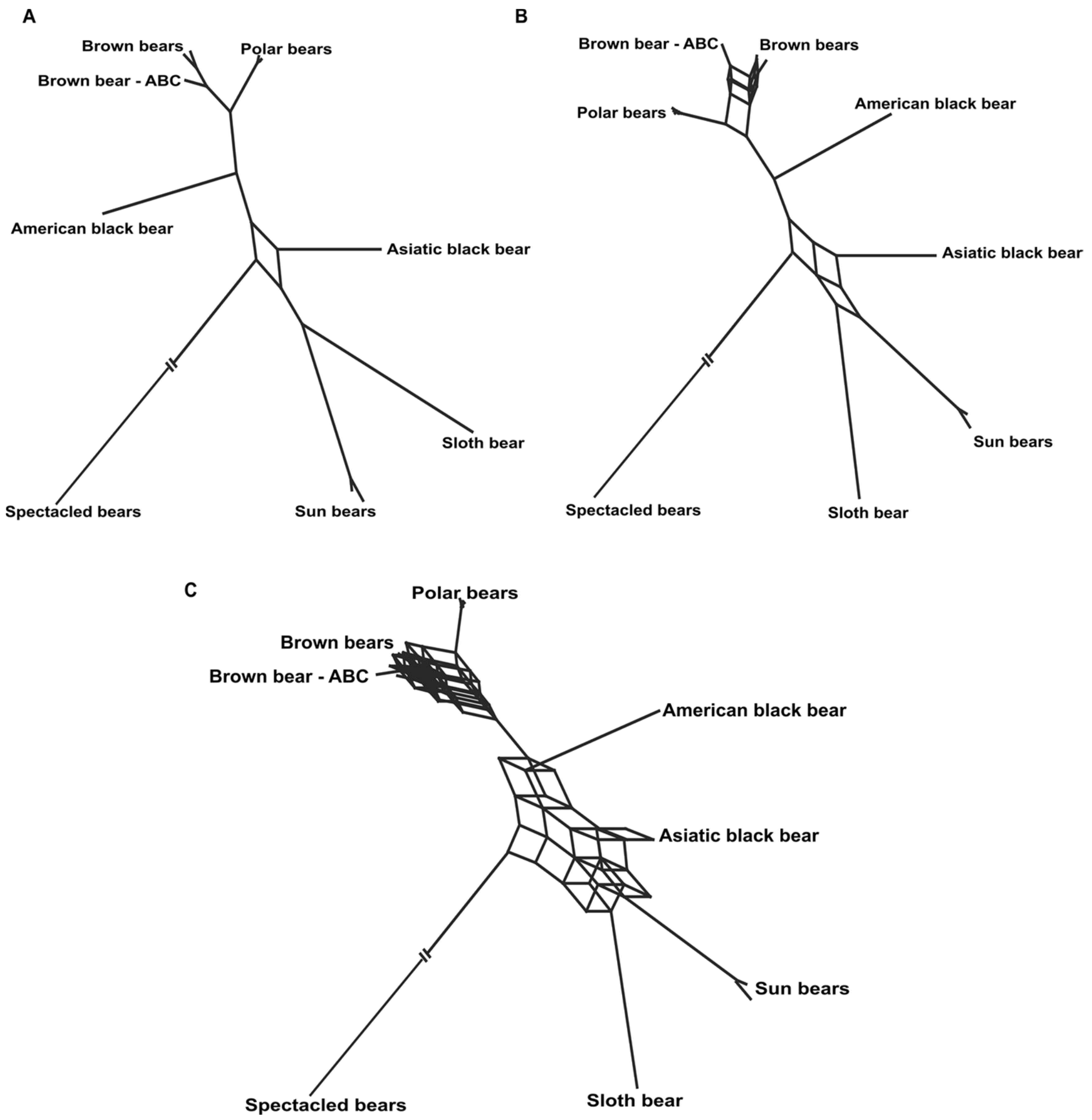

Supplementary Fig. 9. Network analysis of 18,621 GF ML trees. SplitsTree with thresholds at (A) 30%, (B) 10% and (C) 5%. At the 30% threshold the Asiatic black bear is either sister group to the sun and sloth bear, or to the clade of American black, brown plus polar bear. It is evident from the figure that signal is becoming increasingly complex with lower thresholds.

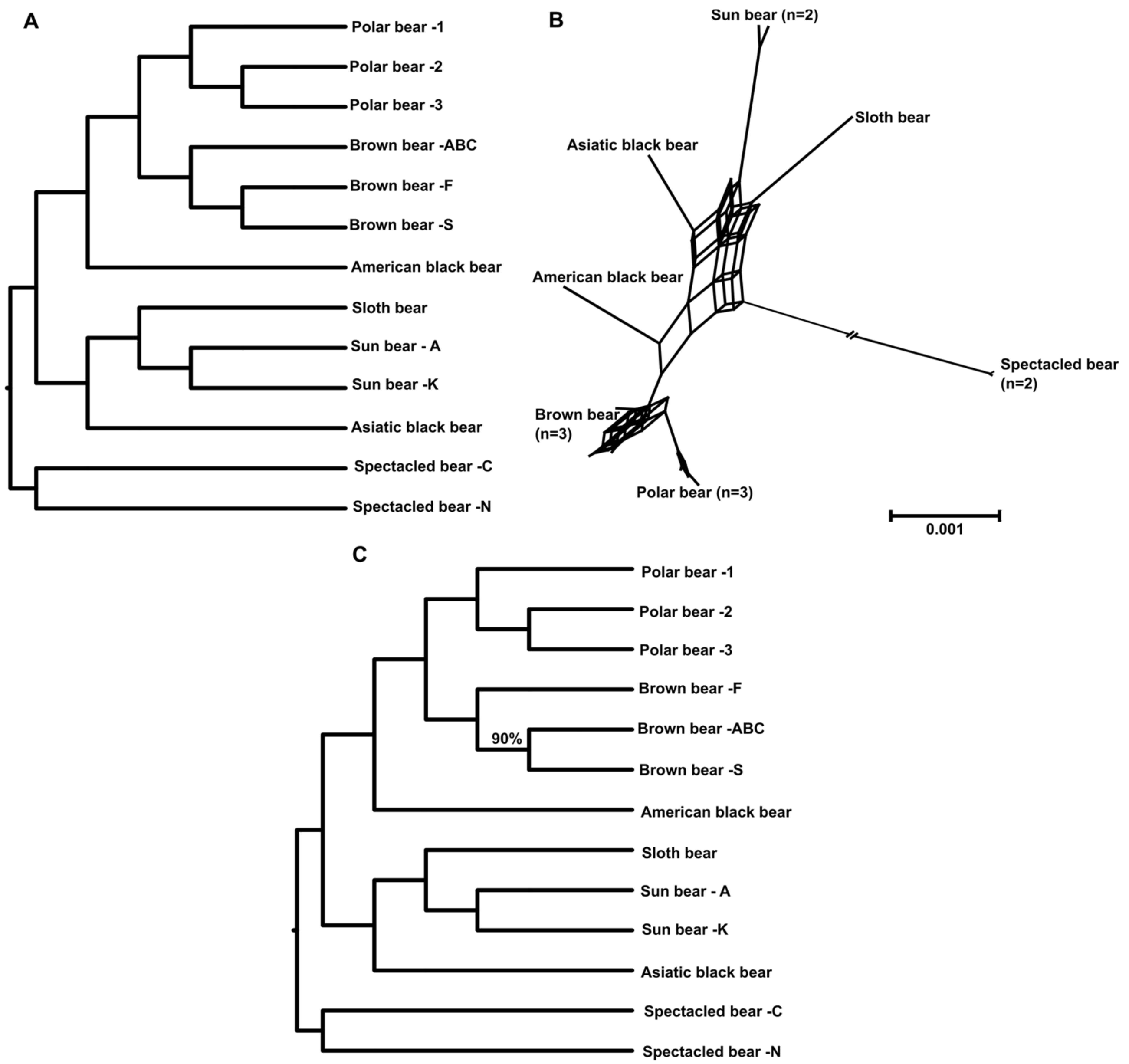

Supplementary Fig. 10. The X chromosome species tree, X chromosome network, and coding sequence species tree. A) A coalescent species tree (cladogram) from 718 GF >1000bp (total 74Mb) was constructed from X chromosome scaffolds identified by [5]. The tree is identical to that of Fig 2A. B) The splits network from X chromosome data with 8% threshold is very similar to that for the whole genome Fig 2B. C) The coalescent species tree from 8,050 protein coding genes (10,303,323 bp). Note that all branches are supported by 100% bootstrap support, except the one placing the ABC-island brown bear. Giant panda as an outgroup is not shown. Brown bear-F: Brown bear -Finland, Brown bear-S: Brown bear -Sweden, Sun-A: Sun bear- Anabell, Sun-K: Sun bear -Klaus, Spectacled bear-C: Spectacled bear- Chappari and Spectacled bear-N: Spectacled bear – Nobody. The scale bar indicates 0.001 substitutions per site.

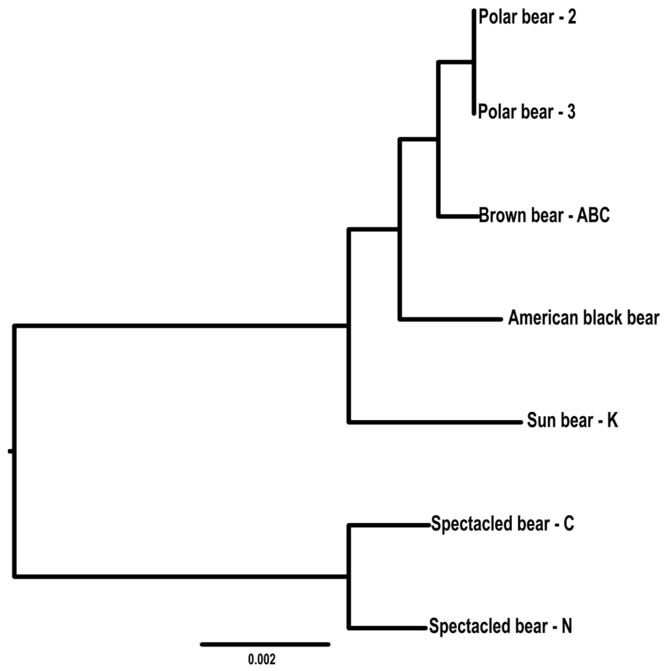

Supplementary Fig. 11. A ML species tree reconstructed from 7.96 kb of Y chromosome scaffolds. The analysis is based on identified Y chromosome scaffolds [6] and all branches receive 100% support. Only scaffolds which are *in vitro* validated or longer than 1 kb: Scaffold ID: 297, 301, 309, 318, 369, 389, 403, 579, 605, 646, 4889 and 6612 [6], were used in the analysis. Names see Supplementary Fig. 10. The scale bar indicates 0.002 substitutions per site.

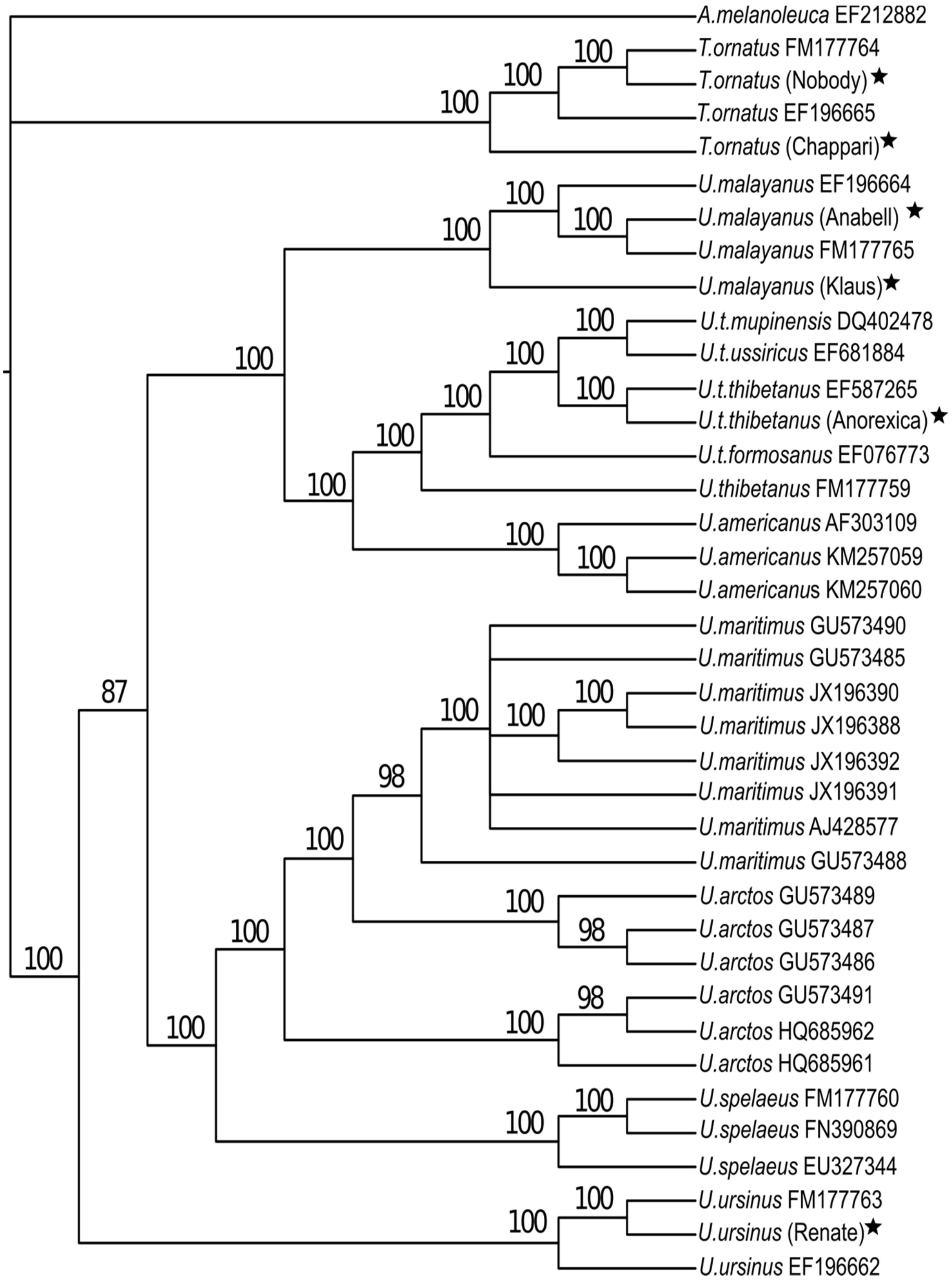

Supplementary Fig. 12. Bayesian tree from complete mitochondrial genomes (11,529 bp alignment) of 38 bears with species name and accession numbers. The values on the branches show the posterior probability values (x100). Binomial names with asterisk represent genomes new to this study with their individual name in bracket. Note the limited support for placing the sloth bears (*U. ursinus*) as sister group to all other ursine bears.

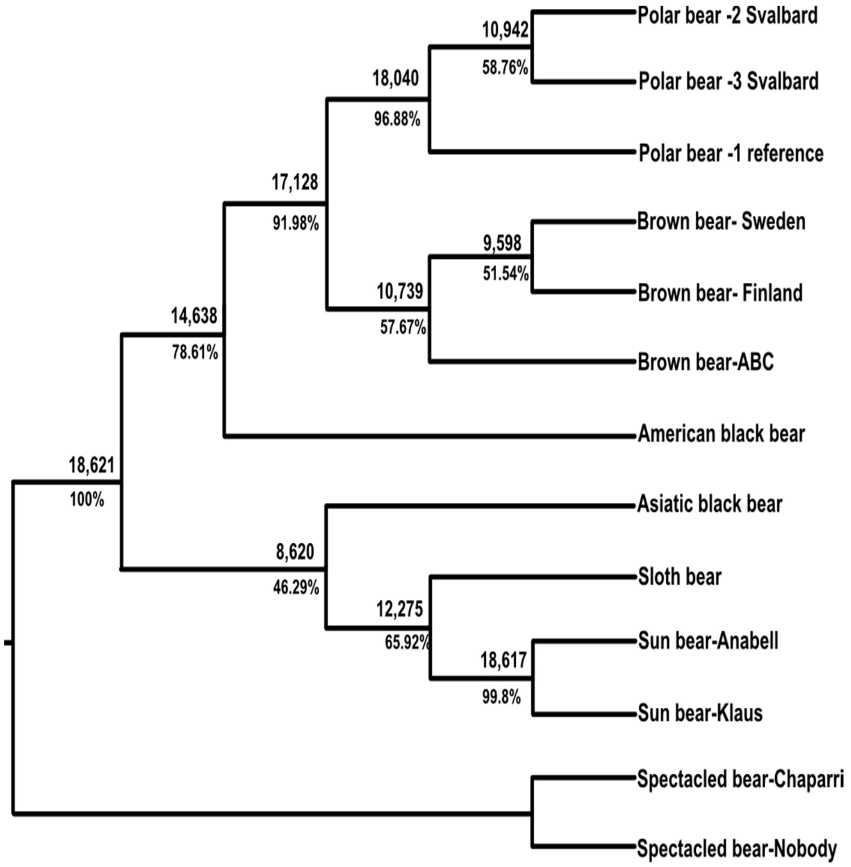

Supplementary Fig. 13. A majority rule consensus tree from 18,621 individual GF ML trees (Supplementary Table 3) calculated with the program consense of the Phylip package. The topology is congruent to the coalescent species tree. Number above each branches indicate the absolute number of splits found in 18,621 individuals GF trees, the number below shows the percentage values. The low support (46.3%) for placing the Asiatic black bear as the sister group to the sun and sloth bear is congruent with the network analysis and gene flow analyses.

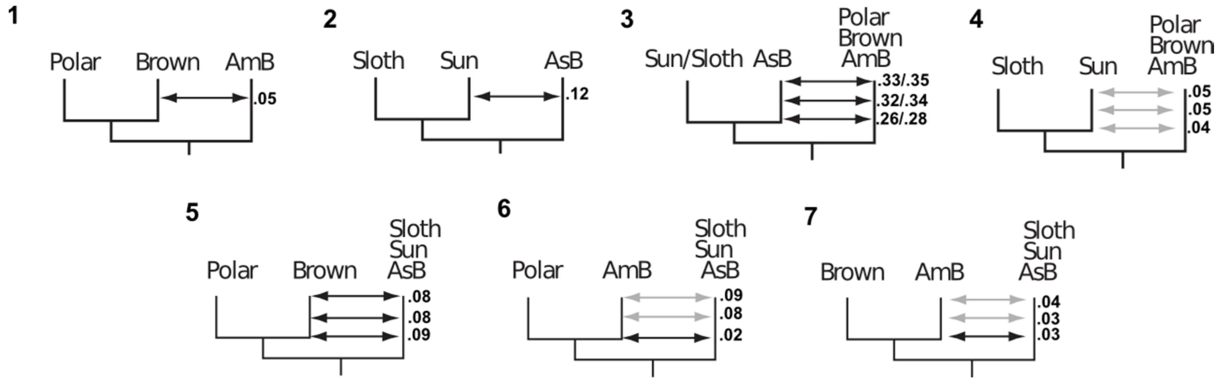

Supplementary Fig. 14. Graphical summary of the D-statistics analyses (Supplementary Table 4). The individual trees show gene flow for different combinations of ursine bears (Polar = Polar bear-1, Brown = Brown bear-Finland, AmB = American black bear, AsB = Asiatic black bear, Sun = Sun bear-Anabell, Sloth = Sloth bear). The *D*-values are shown next to black arrows that symbolize gene flow between the respective species. Gray arrows symbolize possibly indirect or past gene-flow, because the species habitats do currently not overlap, e.g. between American black bear and sun and sloth bear. These species probably never overlapped in space and time, when the American black bear became isolated on the American continent after the divergence of sun and sloth bear. The topology numbers (1-7) is found in Supplementary Table 4.

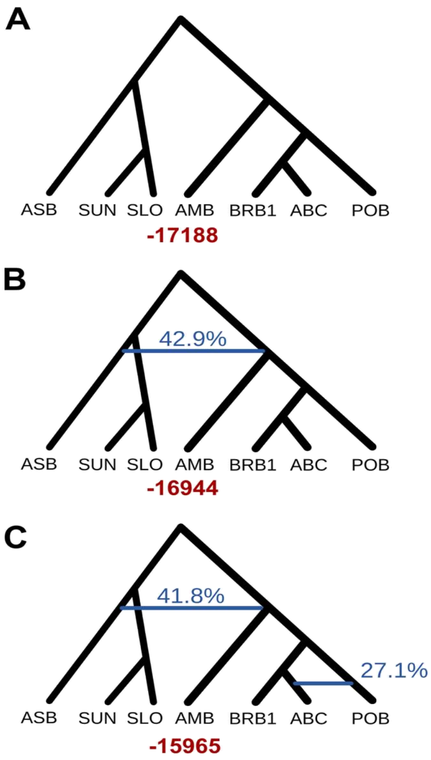

Supplementary Fig. 15. PhyloNet analyses. ML networks for 4000 putatively independent GF trees (every fourth tree) generated by PhyloNet in runs allowing 0, 1 or 2 reticulations (A, B and C). Reticulations are drawn in blue with inheritance probabilities. Exact log-likelihood values were calculated and written in red below each network. The most prominent reticulation between Asiatic black bear and the ancestor of American black, brown and polar bear is also the strongest found in D_{FOIL} analyses. It is obvious that allowing for reticulations (hybridizations) in the networks improves the likelihood values. Only the two most prominent hybridizations are shown, because increasing the number of allowed reticulations increases the computation time from days to months or years. The PhyloNet analyses finds high probability for hybridization between ABC island brown bears and polar bears, confirming earlier observations [7,8]. The prominent gene flow between Asiatic black bear and the ancestor to American black, brown and polar bear is also detected confirming D_{FOIL} analyses (Table 1). PhyloNet probably detects hybridization in particular when gene flow is concentrated in the genome and affects trees from GF, while *D*-statistics detects even a spread-out signal, because it analyses all nucleotide differences in the ABBA/BABA statistics.

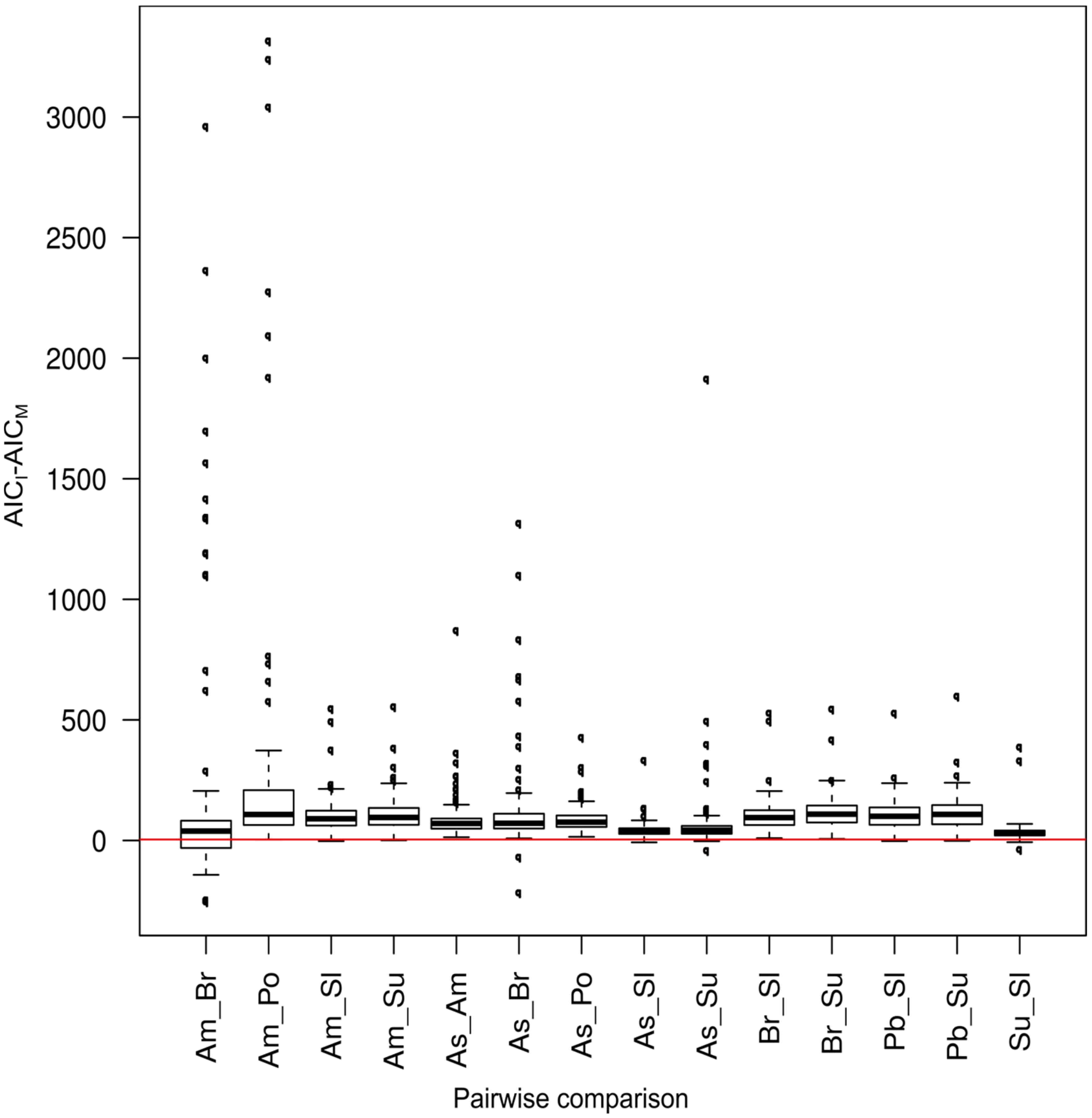

Supplementary Fig. 16. CoalHMM analyses on pairwise species with gene flow.

Box-plots of the distribution of the difference of AIC values under the isolation model and AIC values for the isolation with migration model for different species pairs (separated by _). Positive AIC values show preference for the migration model while negative values favour the isolation model. Many genomic fragments have negative AIC values, but a substantial number of fragments have ΔAICs above zero, thus significantly rejecting the isolation model. Am= American black bear, As = Asiatic black bear, Br = brown bear, Sl = Sloth bear, Sun = Sun bear, Po = polar bear.

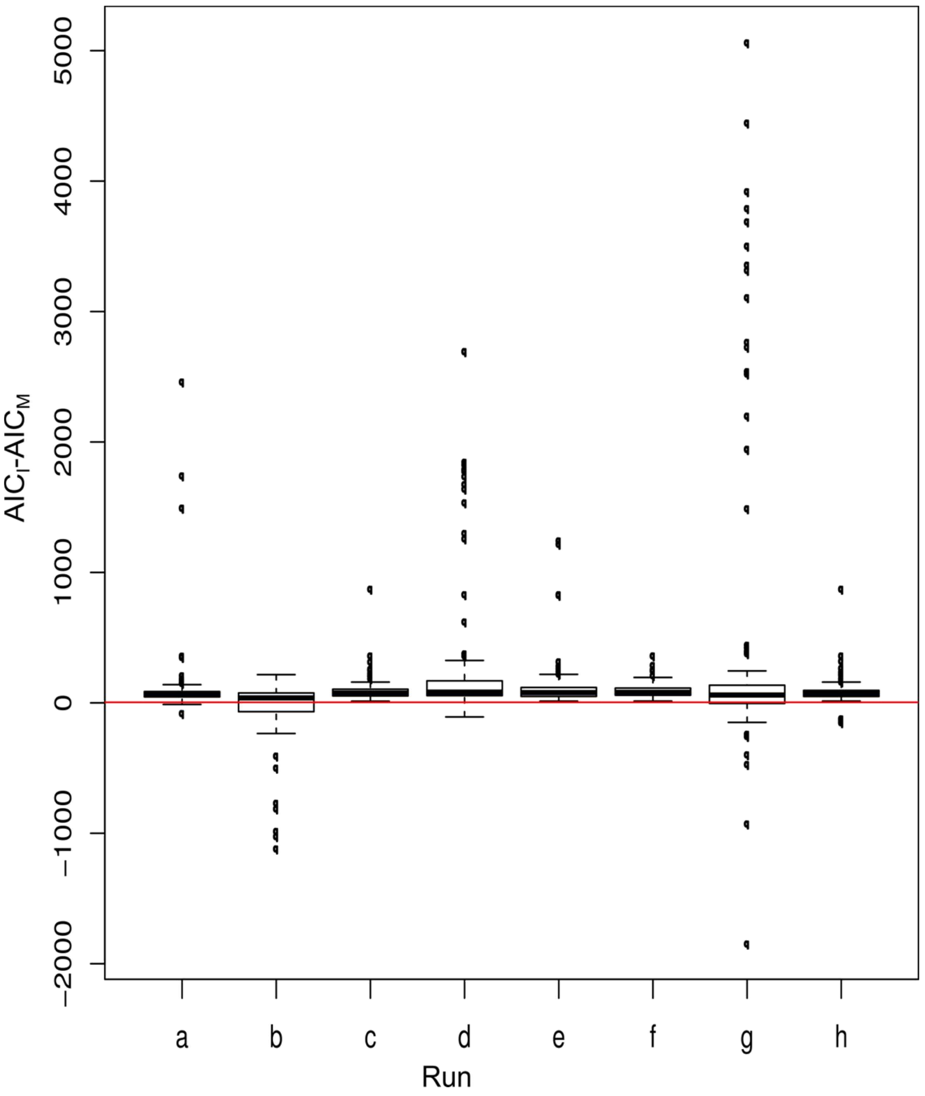

Supplementary Fig. 17. CoalHMM sensitivity analysis. Most population parameters are difficult to precisely estimate, especially for past population sizes. The influence of unrealistically high or low parameters are studied in a sensitivity analyses for the American black bear and Asiatic black bear species pair. (a) N_e 2,000, (b) N_e 200,000, (c) N_a = 5 x N_e, (d) μ = 0.5e-9, (e) μ = 2.0e-9, recombination rate (f) 0.1 and (g) 10 , migration time 8% of split time. The estimates are robust over a broad range of parameters. The largest impact on the analyses, still with many genomic fragments showing a positive signal, came from the (g) recombination rate parameter. This indicates that the result of a migration model is insensitive over a wide range of parameters at least for a substantial part of the genome.

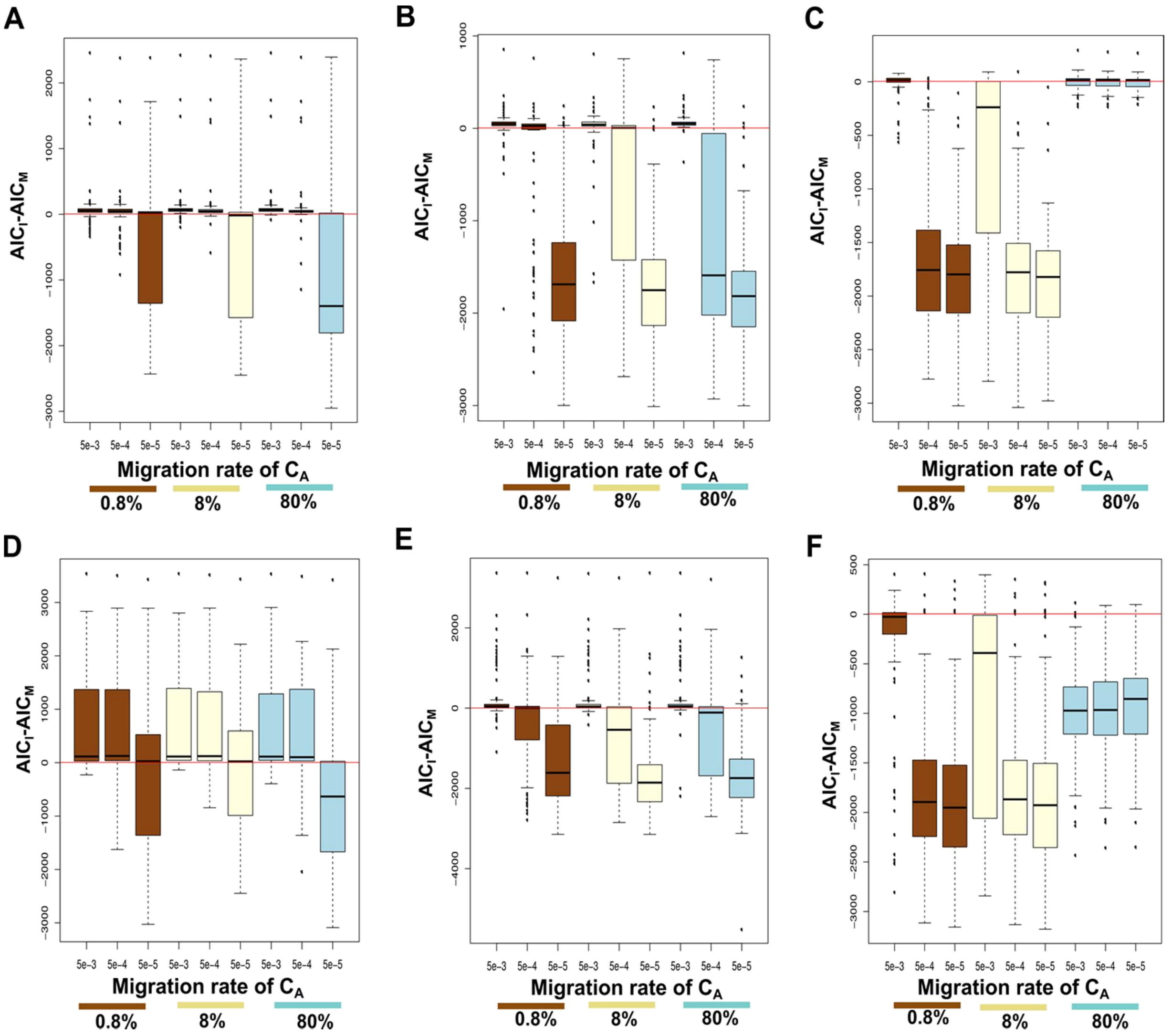

Supplementary Fig. 18. Exploration of different parameters for the CoalHMM analyses. Box plots in A, B and C show the Asiatic and American black bear species pair with migration rate values that are three orders of magnitude lower (0.005, 0.0005 and 0.00005 multiplied C_A) than described for other mammals [9,10]. These values are explored under an *Ne* of 2,000 (A), an *Ne* of 20,000 (B) and *Ne* of 200,000 (C), and migration times of 0.8% (brown), 8% (grey) and 80% (blue) of the split time, other parameters are as described before (Supplementary Fig. 16). Box plots D, E and F show the American and brown bear pairwise comparison with the parameters set as above. It is evident that decreasing the migration rate reduces the number of genomic fragments supporting the migration model. However, even at the lowest migration rates (representing << 0.01 migrant per generation) a non-negligible amount GFs supports the migration model.

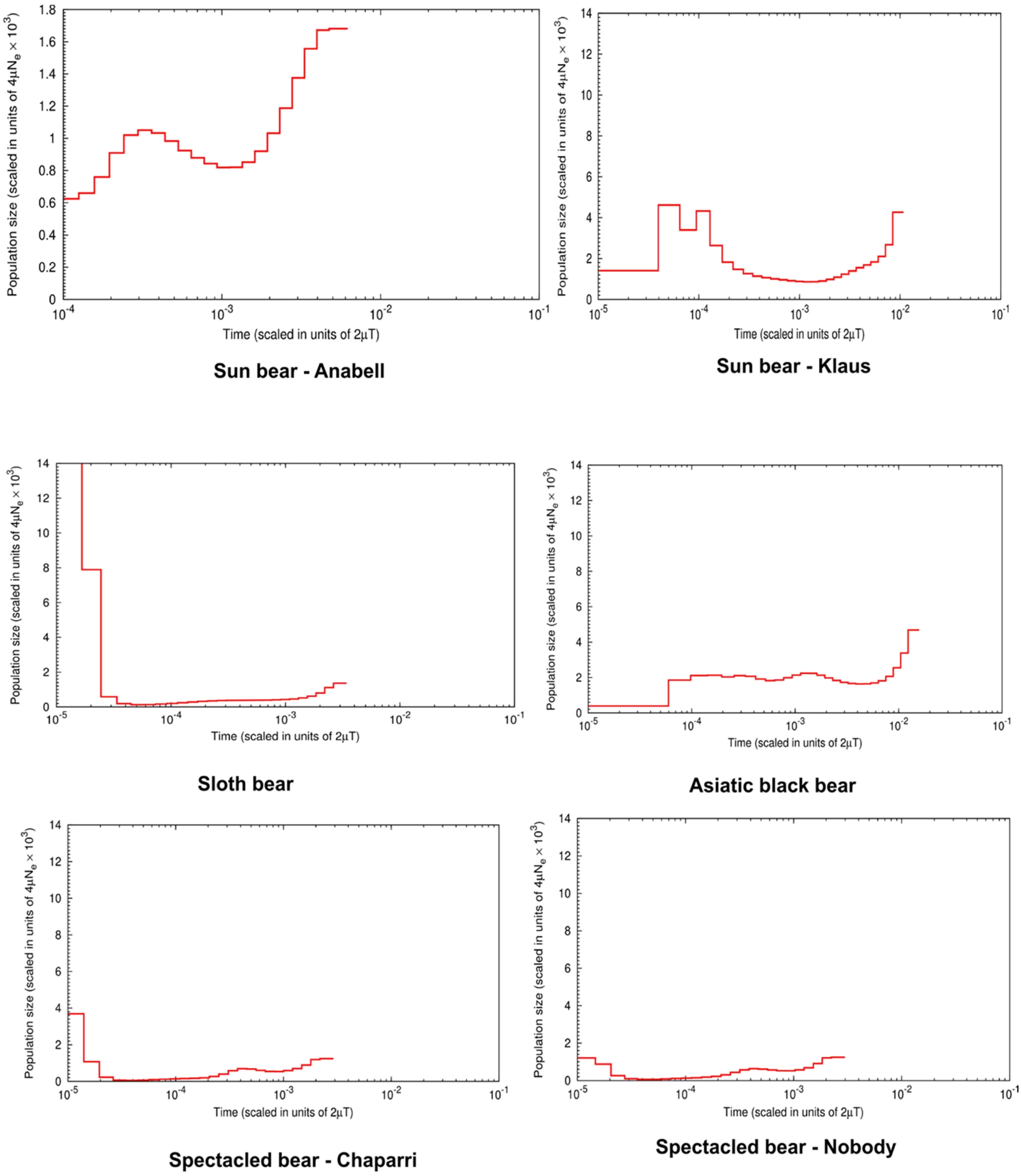

Supplementary Fig. 19. Unscaled pairwise Sequential Markovian Coalescent (PSMC) plots used in this study.

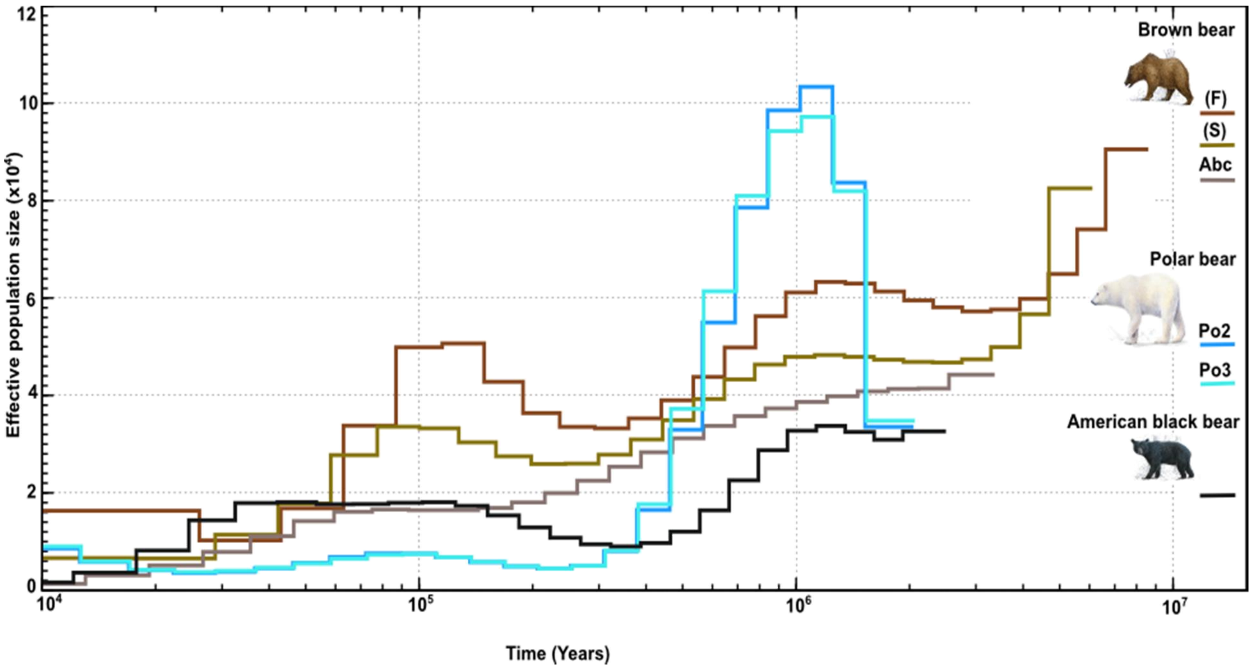

Supplementary Fig. 20. Demographic history from polar, brown and American black bears genomes used in this study. Pairwise Sequential Markovian Coalescent (PSMC) analysis using the mutation rate of 1×10^{-8} changes/site/generation with a generation time of 6 years. The bear paintings were made by Jon Baldur Hlidberg (www.fauna.is).

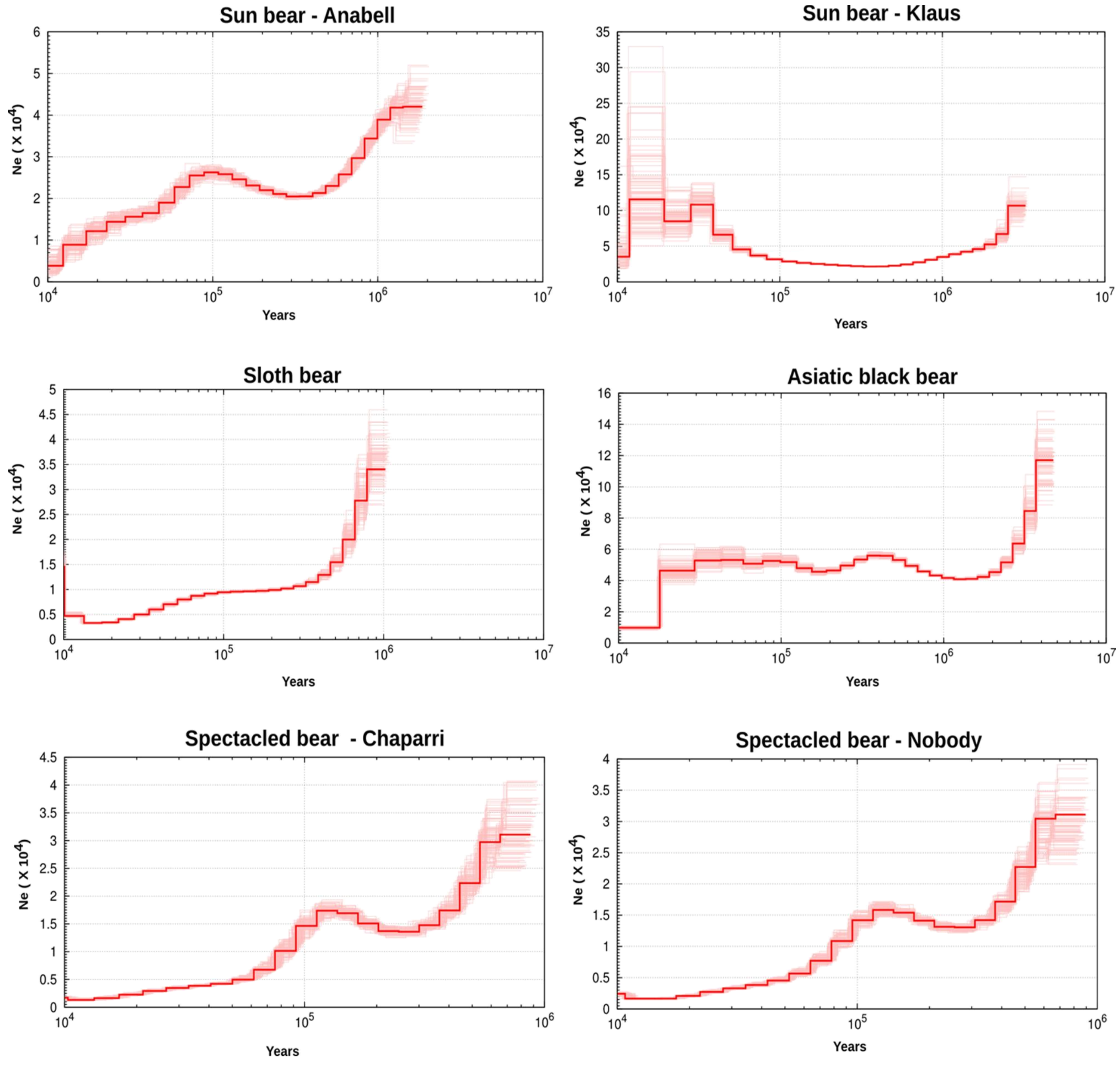

Supplementary Fig. 21. Demographic history of the sequenced bear genomes. Pairwise Sequential Markovian Coalescent (PSMC) analysis using the mutation rate of 1×10^{-8} changes/site/generation with a generation time of 6 years. Light red lines summarize 100 bootstrap replicates from the PSMC analysis.

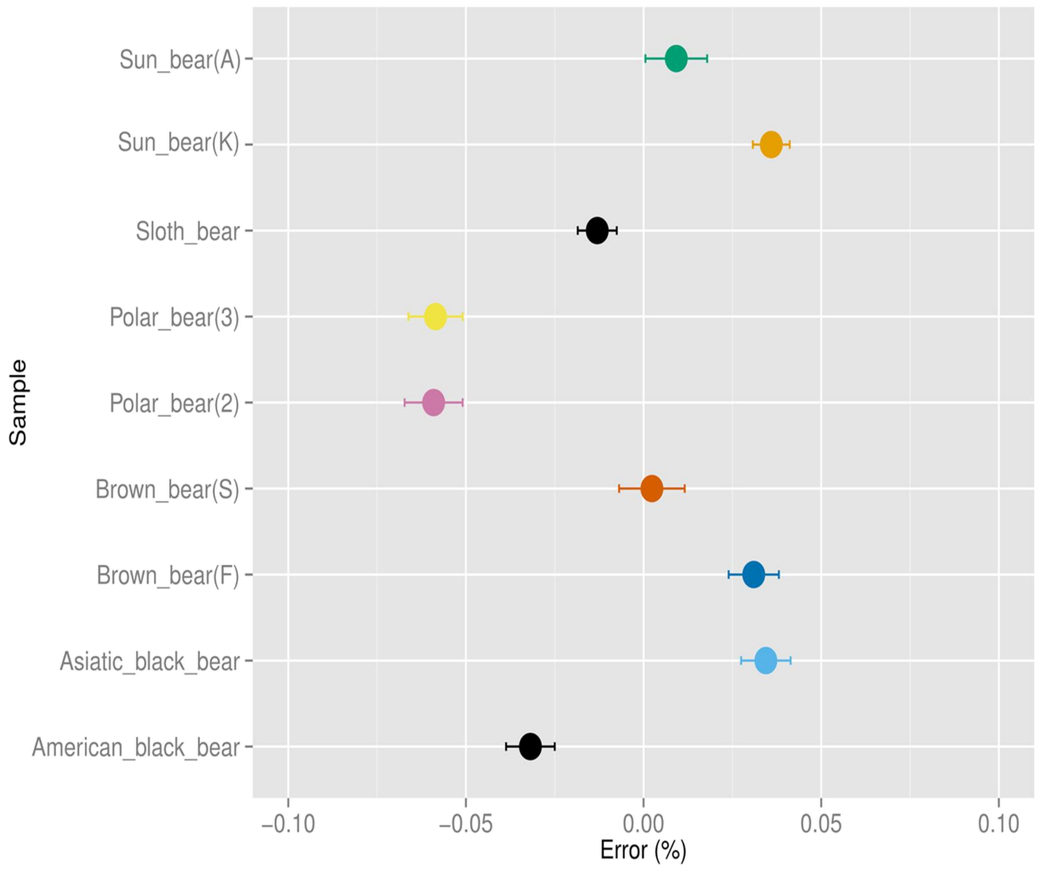

Supplementary Fig. 22. Genome error rate analysis. Mean error percentages for bear genomes calculated on 430 Mb sequence data. Error bars indicate 95% confidence intervals. Note, that depending on the excess of expected derived and expected ancestral alleles, the error rate can be negative or positive.

Supplementary Tables

Supplementary Table 1. Details of the available (published) and newly sequenced bear genomes involved and analyzed in this study. Binomial names with asterisk (*) represent genomes new to this study. Binomial name has been adopted according to reference [11] in this study. F: female, M: male.

Binomial name according to IUCN	Binomial name according to Nowak 1991	Common name and identifier	Sex	SRA number / EBI Accession number	Origin	Individual Name / Stud-book number
Ursus maritimus	*Ursus maritimus*	Polar bear 1	M	n.a. (Reference genome)	n.a.	n.a.
Ursus maritimus	*Ursus maritimus*	Polar bear 2	M	SRR518686, SRR518687	Svalbard	n.a.
Ursus maritimus	*Ursus maritimus*	Polar bear 3	M	SRR518661, SRR518662	Svalbard	n.a.
Ursus arctos	*Ursus arctos*	Brown bear – ABC	M	SRR518717	ABC-island	n.a.
Ursus arctos	*Ursus arctos*	Brown bear	F	SRR935592, SRR935595, SRR935624, SRR935628	Finland	n.a.
Ursus arctos	*Ursus arctos*	Brown bear	F	SRR935591, SRR935625, SRR935627	Sweden	n.a.
Ursus americanus	*Ursus americanus*	American black bear	M	SRR518723	Alaska	n.a.
*Ursus thibetanus**	*Ursus thibetanus*	Asiatic black bear	F	PRJEB9724	Zoo Madrid	Anorexica / 201
*Melursus ursinus**	*Ursus ursinus*	Sloth bear	F	PRJEB9724	Zoo Leipzig	Renate
*Helarctos malayanus**	*Ursus malayanus*	Sun bear	F	PRJEB9724	Zoo Münster	Anabell / T1328
*Helarctos malayanus**	*Ursus malayanus*	Sun bear	M	PRJEB9724	Zoo Madrid	Klaus
*Tremarctos ornatus**	*Ursus ornatus*	Spectacled bear	M	PRJEB9724	Zoo Basel	Chaparri
*Tremarctos ornatus**	*Ursus ornatus*	Spectacled bear	M	PRJEB9724	Zoo Basel	Nobody

Supplementary Table 2. Sequencing and assembly statistics of all the analyzed bear individuals in this study. The columns show the final number of reads used for the assembly with the number of raw and clean reads, the percentage of reads that were mapped, the initial mapping coverage and coverage after mark duplicates, and homo- and heterozygous SNVs sites called relative to polar bear reference genome. Common names with asterisk represent genomes new to this study.

**Common name**	**ID**	**Raw reads (Million)**	**Cleaned reads (Million)**	**Mapped reads (%)**	**Initial coverage (X)**	**Final coverage (mark duplicates) (X)**	**Homozygous SNVs (Million)**	**Heterozygous SNVs (Million)**
Polar bear	2	351.5	336.8	97.4	13.9	13.4	0.6	0.7
Polar bear	3	358.1	343.4	96.1	13.8	13.5	0.6	0.7
Brown bear	Finland	686.3	637.6	95.7	23.9	22.3	5.0	3.9
Brown bear	Sweden	669.5	615.3	74.0	18.1	15.8	4.9	2.7
Brown bear-ABC	ABC-island	1122.1	1022.1	97.3	38.7	29.1	3.8	1.3
American black bear	Alaska	891.8	832.2	96.0	30.7	21.1	8.4	1.1
Asiatic black bear*	Anorexica	338.9	317.9	95.9	11.4	11.0	9.4	4.1
Sloth bear*	Renate	301.7	285.9	96.0	10.5	9.9	13.2	0.8
Sun bear*	Anabell	301.1	286.0	95.6	10.4	10.1	12.8	1.3
Sun bear*	Klaus	328.6	311.0	96.0	11.3	10.9	12.1	2.6
Spectacled bear*	Chaparri	325.4	307.2	96.2	11.1	10.8	27.1	0.5
Spectacled bear*	Nobody	319.2	301.3	96.3	10.9	10.5	27.0	0.5

Supplementary Table 3. A majority rule consensus analysis of 18,621 individual GF ML-trees. Only splits occurring more than 1% are shown.

Splits included in the consensus tree		
Ranking	Splits (species in order)	Number of times occurring
1	*********. .**	18621
2	....**	18617
3	.....***.. ...	18040
4	...******. ...	17128
5	..*******. ...	14638
6	*......... .**	12275
7	.....*.*.. ...	10942
8	...**...*. ...	10739
9	...**..... ...	9598
10	**........ .**	8620
Splits NOT included in consensus tree		
1	.********. ...	7086
2	.....****. ...	4683
3	.....**... ...	3844
4	....*...*. ...	3785
5	......**.. ...	3616
6	.*........ .**	3474
7	...*....*. ...	3023
8	.*.******. ...	1609
9	**........ ...	1394
10	...*.****. ...	1188
11	...*****.. ...	1109
12	....*****. ...	1096
13	...*.***.. ...	959
14	.********. .**	954
15	***....... .**	904
16	....****.. ...	769
17	*.*******. ...	658
18	.**....... ...	642
19	*********. ...	621
20	**.******. .**	515
21	..*..***.. ...	503
22	..***...*. ...	480
23	*.*....... .**	467
24	..*******. .**	299
25	*.*....... ...	246
26	.....*.**. ...	212

Note – The table summarizes the results from the consense analysis [12]. The ranking is according to the number of occurrences of splits. Only splits occurring more frequent than 1% are shown. In each vertical column dots (.) and asterisks (*) represents one individual and its split into the respective group (. or *). The species order in the row of dots (.) and asterix (*) is as follows:1st Sloth bear, 2nd Asiatic black bear, 3rd American black bear, 4th Brown bear-Sweden, 5th Brown bear-Finland, 6th Polar bear-1, 7th Polar bear-2, 8th Polar bear-3, 9th Brown bear-ABC, 10th Spectacled bear-Nobody,11th Spectacled bear-Chaparri, 12th Sun bear-Anabell, 13th Sun bear-Klaus. For example: row one

(*********. .**) has species 10 (spectacled bear Nobody) and species 11 (spectacled bear-Chaparri) as the most frequent split (..) against all others (************), row two (.......... .**) has species 12, (sun bear-Klaus) plus species 13 (sun bear-Anabell) as the second most frequent split (**) with 18617 occurrences. One can deduce that in four occurrences they have not been place together, but one of them grouped with another individual. This is not shown, because such an occurrence was less than 1%. The total number of splits exceeds 400. Row 3 groups the three polar bears (***), row 4 shows the split of of the the polar bears plus two of the brown bears, and so on. This way all bifurcations (splits) are shown. Splits that occur less often than 50% are not shown in Supplementary Fig.13. As such, a split that shows the Asiatic black bear plus the American black bear, the brown and polar bears is not shown, but there is phylogenetic signal from 7,086 GF from ILS or geneflow for this grouping (see first row “Splits NOT included in consensus tree”). This is consistent with with the unstable placement of the Asiatic black bear in most other analyses and strong geneflow that is detected between them.

Supplementary Table 4. Significant *D*-statistic values for the selected bear species using the spectacled bear as outgroup. All combinations were considered, with ABBA-BABA counts, *D*-statistics ratio with jackknife estimates, standard error and Z-Score (significant if > |3|). It is evident that we find significant gene flow between all the bear species. The triplets for which gene flow is indicated are numbered (#) and depicted in supplementary fig. S14. In all other cases comparisons are made that are in conflict with the species tree, thus showing a phylogenetic rather than a gene-flow signal. A negative value of *D*-statistics shows that H1 is closer to H3 than H2 is and a positive value shows that H2 is closer to H3 than H1 is.

#	H1	H2	H3	nABBA	nBABA	Dstat	jackEst	SE	Z
1	Polar bear	Brown bear	American black bear	638,073	581,523	0.046	0.046	0.00280	16.575
2	Sun bear	Sloth bear	Asiatic black bear	817,080	1,054,339	-0.127	-0.127	0.00261	-48.523
3	Sloth bear	Asiatic black bear	Brown bear	1,399,921	685,682	0.342	0.342	0.00237	144.417
3	Sloth bear	Asiatic black bear	Polar bear	1,347,365	653,305	0.347	0.347	0.00241	143.651
3	Sun bear	Asiatic black bear	Brown bear	1,307,028	668,618	0.323	0.323	0.00238	135.969
3	Sun bear	Asiatic black bear	Polar bear	1,255,985	637,545	0.327	0.327	0.00243	134.171
3	Sloth bear	Asiatic black bear	American black bear	1,321,219	736,652	0.284	0.284	0.00249	113.880
3	Sun bear	Asiatic black bear	American black bear	1,232,896	716,199	0.265	0.265	0.00246	107.680
4	Sun bear	Sloth bear	Polar bear	686,930	761,829	-0.052	-0.052	0.00223	-23.227
4	Sun bear	Sloth bear	Brown bear	716,312	791,448	-0.050	-0.050	0.00219	-22.716
4	Sun bear	Sloth bear	American black bear	731,653	799,050	-0.044	-0.044	0.00221	-19.880
5	Polar bear	Brown bear	Asiatic black bear	590,580	492,405	0.091	0.091	0.00204	44.412
5	Polar bear	Brown bear	Sloth bear	496,853	419,398	0.085	0.085	0.00223	37.843
5	Polar bear	Brown bear	Sun bear	506,198	427,910	0.084	0.084	0.00231	36.216
6	Polar bear	American black bear	Sun bear	869,012	731,739	0.086	0.086	0.00240	35.728
6	Polar bear	American black bear	Sloth bear	857,657	712,872	0.092	0.092	0.00239	38.516
6	Polar bear	American black bear	Asiatic black bear	944,040	907,709	0.020	0.020	0.00207	9.487
7	American black bear	Brown bear	Sloth bear	765,121	832,022	-0.042	-0.042	0.00241	-17.368
7	American black bear	Brown bear	Asiatic black bear	973,063	910,762	0.033	0.033	0.00212	15.636

7	American black bear	Brown bear	Sun bear	785,215	843,628	-0.036	-0.036	0.00243	-14.733

Note – Polar bear is Polar bear-2, Brown bear is brown bear-Finland, Sun bear is Sun bear-Anabell

Supplementary Table 5. Significant *D*-statistics among ABC brown and polar and also between sloth and sun bear. A negative value of *D*-statistics shows that H1 is closer to H3 than H2 is and a positive value shows that H2 is closer to H3 than H1 is. Z-Score is significant if > |3|.

H1	H2	H3	nABBA	nBABA	Dstat	jackEst	SE	Z
Brown bear- Finland	Brown bear -ABC	Polar bear-2	761,149	541,802	0.168	0.168	0.004772	35.271
Sun bear -Anabell	Sun bear- Klaus	Sloth	144,730	147,578	-0.010	-0.010	0.002690	-3.622

Supplementary Table 6. Significant D-statistics for the selected bear species using the giant panda as outgroup. A negative value of D-statistics shows that H1 is closer to H3 than H2 is. Z-Score is significant if > |3|.

H1	H2	H3	nABBA	nBABA	Dstat	jackEst	SE	Z	*Z (Spectacled bear as out group)*
Sun bear	Sloth bear	Asiatic black bear	1,152,717	1,415,513	-0.102	-0.102	0.001204	-84.953	*-48.523*
Sloth bear	Asiatic black bear	Sun bear	1,415,513	1,569,988	-0.052	-0.052	0.001361	-37.990	*-27.008*
Sun bear	Asiatic black bear	Sloth bear	1,152,717	1,569,988	-0.153	-0.153	0.001293	-118.492	*-79.801*

Note – Sun bear is Sun bear-Anabell

Supplementary Table 7. Divergence time estimates in Ma using the MCMC tree program in PAML based on 5.2 million bp coding sequences.

Splits	Estimated Years (Ma)
Spectacled bear / Ursinae	10.6 (6.7-13.0)
Polar + brown + American black bear / Asiatic black bear + Sun + Sloth bear	5.0 (4.5-6.0)
Asiatic black bear / Sun + Sloth bear	4.4 (3.6-5.8)
Sun / Sloth bear	3.6 (2.4-5.6)
American black bear / Polar + Brown bear	3.4 (2.0-4.7)
Polar / Brown bear	0.9 (0.6-1.1)
Brown bear-ABC /Brown bear-F	0.5 (0.4-0.8)
Sun bear / Sun bear	0.7 (0.2-0.8)
Brown bear-S/Brown bear-F	0.3 (0.2-0.5)
Polar bear-1 / Polar bear-2	0.1 (0.1-0.3)
Polar bear-2 /Polar bear-3	0.1 (0.0- 0.2)

Supplementary Methods

Topology testing

The simulated sequences were produced by Seq-Gen version 1.3.3 [13] using the species tree topology (Fig 2A) and ML branch lengths based on 10 Mb of random genomic data with parameters (Settings: -mGTR -g4 -i0.1 -n100). Increasingly longer simulated sequences were produced, starting at 300 bp, and using 300 bp increments for sequences with lengths of up to 40,500 bp. Five different topologies were selected for statistical evaluation using the two different methods of simulation and real genomic data sets analyses (Supplementary Fig. 6). The sequences were analyzed by using the RAxML version 8.2.4 [14] and AU probabilities were calculated using CONSEL version 1.20 [15] using the GTR+G+I [16] model of sequence evolution. The best fitting substitution model was estimated using the jModelTest 2.1.1 [17] on 10 Mb of random GFs available in RAxML version 8.2.4 [14]. A second AU analysis was done on real genomic data by selecting 500 random GF that support the coalescent species tree (Fig 2A) as the best tree. The amount of substitutions that were contained in each GF was evaluated to make sure that there was sizeable genetic distance between the species for phylogenetic analysis.

In addition, to calculate the range of pairwise uncorrected genetic distance in the filtered GFs, pairwise uncorrected genetic distances between the three Asiatic bear species were calculated using custom perl scripts. The removal of TEs and simple repeats from the GF resulted in alignments of varying lengths, ranging between <5,000 to 80,000 nt (Supplementary Fig. 2). Model testing determined the GTR+G+I model of sequence evolution as the best fitting model available in RAxML version 8.2.4 [14]. It was used in all subsequent phylogenetic ML analyses. The AU likelihood statistics of simulated GF sequences indicate that only alignments with a length >25 kb contain sufficient phylogenetic information to reject alternative trees. The mtDNA tree, which is the most different compared to the coalescent species tree, is significantly rejected already with 4,500

bp sequence length of nuclear DNA (Supplementary Fig. 6). Topologies that deviate less from the coalescent species tree, in particular those placing the Asiatic bear species in different positions requires sequence lengths of about 25 kb to be rejected. For that reason, data sets shorter than 25 kb were discarded from further phylogenetic analysis.

For natural reasons simulated data represent ideal datasets that are largely free of noise such as, ILS or gene flow. Therefore, these simulations define a lower bound of sequence length that is needed to reject alternative hypotheses. As a consequence, the selected size of the GFs represents a compromise to obtain sufficient phylogenetic information while still being short enough to minimize mixed phylogenetic signals from recombination. The typical size of non-recombining haplotype blocks is not yet known for the bear genomes, however it may be expected from the smaller effective population size in bears that it is larger than 11-22 kb as observed in humans [18]. The assumption that 25 kb contains enough phylogenetic signal, was further evaluated on 500 real GFs. The evaluation of real data with a length of 25,000 bp shows that most alternative topologies are significantly rejected by a AU analysis (Supplementary Fig. 6). However, it is evident from the plot that the GFs cannot reject all the topologies, especially topology 4 and topology 5 (Supplementary Fig. 6) which differ only little from the species tree (Fig 2A). Thus, these GFs may still contain a mixed phylogenetic signal, which favors another alternative topology.

For resolving evolutionary questions, the amount of phylogenetic information (substitutions) per GF is crucial. The average number of 104 bp simple pairwise differences between the three Asiatic bears in 25-30 kb long GFs, gives an idea about the typical phylogenetic signal in GFs (Supplementary Fig. 5). Thus, these analyses show that GF of >25 kb contain sufficient phylogenetic signal to discriminate between topologies. This in stark contrast to the 1 kb fragments that were used in a recent study on primates (gibbons) where high recombination is known to have taken place [19].

Phylogenetic analysis of nuclear protein-coding genes and mitochondrial genomes

The annotation of the polar bear genome [20] was used to extract the protein coding sequences (CDS) from the genome that could be used for phylogenetic analysis. The species alignments were complemented by giant panda (*Ailuropoda melanoleuca*, ailMel1) sequences from Ensembl (http://www.ensembl.org/) to provide an outgroup to the analyses. In order to determine orthologous CDS between the polar bear reference genome and the giant panda Proteinortho version 5.06 [21] was used. The CDS of the new bear genomes that corresponded to the polar bear CDS were aligned with MAFFT version 7.154b [22]. Gaps were removed using Gblocks version 0.91b [23] and a custom perl script removed ambiguous sites. CDS <300 bp were not used for phylogenetic analyses. The best evolutionary model, GTR+G+I [16], was estimated using jModelTest 2.1.1[17]. A coalescent species tree was constructed with bootstrap support with Astral [24] from individual CDS using the GTR+G+I model of sequence evolution. In addition, a concatenated analysis of the coding sequence was also done to estimate the concatenated CDS tree. The CDSs were concatenated and the substitution model GTR+G+I was used to create an ML tree with RaxML [14]. A AU topology test was made on the CDS topology using the CONSEL version 1.20 [15] and the species tree (Fig. 2A).

In order to extract the complete mt genomes from Illumina sequence data, the reads for different bear species were mapped to their respective published complete mt genome sequences using BWA version 0.7.5a [25]. Consensus sequences were created using Samtools version: 0.1.18 [26], aligned by MAFFT version 7.154b [22] to 32 published sequences (Accession numbers see Supplementary Fig. 12), and MrBayes version 3.2.2 [27] was used to create the Bayesian phylogenetic tree using the best fitting GTR+G+I model of sequence evolution. The analysis was run for 4,000,000 generations with a sample frequency of 4,000 with default priors and an arbitrary burn in of 25% of the samples. Convergence was assessed using the average standard deviation of split frequency which reached < 0.01 and potential scale reduction factor close to 1.00.

Divergence time estimation

A well-documented fossil from the giant panda lineage at 12 million years ago (Ma) [28] with a maximum calibration point of 20 Ma based on mitochondrial estimates [29] was used to provide the calibration point needed in PAML MCMCtree [30] to estimate divergence times on 5,151,660 bp coding sequence data. In addition, the divergence time of Tremarctinae was set to 7-13 Ma [31]. Divergence time estimates of Ursinae was based on the occurrence of *U. minimus* at 4.3-6 Ma [32], and a polar/brown bear divergence was given a range of 0.48-1.1 Ma [7,20,33]. The calibration points were used to estimate divergence times in MCMC tree in PAML [30] with a sample size of 2,000,000, burn-in of 200,000, and tree sampling every second iteration. Convergence was checked by repeating the analysis again.

Genome error estimation

Following a approach described by [34] the genome error rate was estimated in 3-way alignment including the sample genome, a high-quality genome and a genome representing ancestral states. We considered the genome sequence of the brown bear (ABC) as high-quality given a 38X coverage, which was the highest among our sampling. The genome sequence of the spectacled bears represents the ancestral state in the alignment. The test assumes the same evolutionary distance between sample and the high quality genome. As errors in the high quality genome never can be ruled out, the error rate is the excess error relative to the high quality genome.

The genome error ε is defined by the equation:

$$OD = ED\,(1-\varepsilon) + EA\,\varepsilon \qquad (1)$$

solved for ε

$$\varepsilon = (OD - ED) / (EA - ED) \qquad (2)$$

with

OD, observed number of derived sites in the sample

ED, expected number of derived sites

EA, expected number of ancestral sites in the sample.

Error rates were calculated for 67 Mb of the genome (scaffold1).

We report very low error rates between -0.00058 and 0.00037. The estimates are in the range of error rates as reported for equid genomes [34].

CoalHMM analysis

CoalHMM [9] was used to estimate whether a pair of species showing gene flow in *D*-statistic and D_{FOIL} analyses, diverged in allopatry or in sympatry with gene flow. In this analysis pair wise Akaike Information Criteria (AIC) [35] values from the isolation model and isolation with migration model are compared on the basis of different population parameters. We selected 10 Mb of non-overlapping genomic fragments and set the effective population size (N_e) to 20,000 for all bears, which represents approximately an average estimate by the PSMC analyses (Fig 4, Supplementary Fig. 20), removing extreme values. The time for species splits was set according to estimated divergence times (Supplementary Table 7). These values seem more reliable then estimating divergence times from population splits shown in PSMC analyses (Fig 6, Supplementary Fig. 19). The generation time (g) was set to 8 years, which is a reasonable average of the published values for large and small-bodied bears [36]. The mutation rate was set to μ = 1e-9 changes/site/year which is common rate in mammals [8,37,38] and the coalescent rate was determined to $(g \cdot \mu \cdot N_a)^{-1} = 2{,}500$ with N_a being the ancestral effective haploid population size 50,000. N_a can be calculated from N_e [39].The migration rate was set to 0.05 of the coalescent rate similar (C_A) to previous publications which is equal to the which equals 0.1 migrants per generation (*Ne*m) [9,10]. The recombination rate was set to 1, which is a lower average from published observed values in carnivores and is typical

for mammals for which 0.5-1.1 cM/Mb are observed [40]. The migration time was 80% of the divergence time. Population parameters vary over time and some values are impossible to pin-point. Therefore, we analyzed the parameter space for the American black and Asiatic black bear species along with American black bear and brown bear species pair over a large parameter space. For details see the Supplementary Fig. 17 and 18 figure legends. All analyses clearly favored the migration model for the American black and Asiatic black bear as well as the brown and American black bear species pair. Some values are chosen to be unreasonably extreme for demonstrating the robustness of the data and conclusions for different settings.

X and Y chromosome (scaffold) tree

Known X chromosome scaffolds [5] have been used to construct the coalescence species tree and phylogenetic network as described for the GFs analyses. A ML tree was constructed from concatenated Y chromosome scaffolds [41], because of the non-recombining nature of the most of the Y-chromosome.

References

1. Kolter, L. European Sun Bear Studbook, Cologne zoo. (2013).
2. Krakowiak, M. European Asiatic Black Bear Studbook, Warsaw zoo. (2013).
3. Kolter, L. European Spectacled Bear Studbook, Cologne zoo. (2014).
4. Langguth, S. European Sloth Bear Studbook, Leipzig zoo. (2014).
5. Cahill, J. A. *et al.* Genomic Evidence for Island Population Conversion Resolves Conflicting Theories of Polar Bear Evolution. *PLoS Genet.* **9,** e1003345 (2013).
6. Bidon, T. *et al.* Brown and polar bear Y chromosomes reveal extensive male-biased gene flow within brother lineages. *Mol. Biol. Evol.* **31,** 1353–1363 (2014).
7. Hailer, F. *et al.* Nuclear genomic sequences reveal that polar bears are an old and distinct bear lineage. *Science* **336,** 344–347 (2012).
8. Miller, W. *et al.* Polar and brown bear genomes reveal ancient admixture and demographic footprints of past climate change. *Proc. Natl. Acad. Sci.* **109,** E2382–E2390 (2012).
9. Mailund, T. *et al.* A New Isolation with Migration Model along Complete Genomes Infers Very Different Divergence Processes among Closely Related Great Ape Species. *PLoS Genet* **8,** e1003125 (2012).
10. Jónsson, H. *et al.* Speciation with gene flow in equids despite extensive chromosomal plasticity. *Proc. Natl. Acad. Sci. U. S. A.* **111,** 18655–18660 (2014).
11. Nowak, R. *Walker's Mammals of the World*. (Johns Hopkins Press, 1991).
12. Felsenstein, J. PHYLIP (Phylogeny Inference Package) version 3.6 *Available from: Author Department of genome sciences, University of Washington. Seattle*. (2005)
13. Rambaut, A. & Grassly, N. C. Seq-Gen: an application for the Monte Carlo simulation of DNA sequence evolution along phylogenetic trees. *Comput. Appl. Biosci. CABIOS* **13,** 235–238 (1997).
14. Stamatakis, A. RAxML Version 8: A tool for Phylogenetic Analysis and Post-Analysis of Large Phylogenies. *Bioinformatics* **30,** 1312-1313 (2014).
15. Shimodaira, H. & Hasegawa, M. CONSEL: for assessing the confidence of phylogenetic tree selection. *Bioinformatics* **17,** 1246–1247 (2001).
16. Lanave, C., Preparata, G., Sacone, C. & Serio, G. A new method for calculating evolutionary substitution rates. *J. Mol. Evol.* **20,** 86–93 (1984).

17. Darriba, D., Taboada, G. L., Doallo, R. & Posada, D. jModelTest 2: more models, new heuristics and parallel computing. *Nat. Methods* **9,** 772 (2012).

18. Gabriel, S. B. *et al.* The structure of haplotype blocks in the human genome. *Science* **296,** 2225–2229 (2002).

19. Carbone, L. *et al.* Gibbon genome and the fast karyotype evolution of small apes. *Nature* **513,** 195–201 (2014).

20. Liu, S. *et al.* Population genomics reveal recent speciation and rapid evolutionary adaptation in polar bears. *Cell* **157,** 785–794 (2014).

21. Lechner, M. *et al.* Proteinortho: detection of (co-)orthologs in large-scale analysis. *BMC Bioinformatics* **12,** 124 (2011).

22. Katoh, K., Misawa, K., Kuma, K. & Miyata, T. MAFFT: a novel method for rapid multiple sequence alignment based on fast Fourier transform. *Nucleic Acids Res.* **30,** 3059–3066 (2002).

23. Talavera, G. & Castresana, J. Improvement of phylogenies after removing divergent and ambiguously aligned blocks from protein sequence alignments. *Syst. Biol.* **56,** 564–577 (2007).

24. Mirarab, S. *et al.* ASTRAL: genome-scale coalescent-based species tree estimation. *Bioinformatics* **30,** (2014).

25. Li, H. & Durbin, R. Fast and accurate short read alignment with Burrows-Wheeler transform. *Bioinforma. Oxf. Engl.* **25,** 1754–1760 (2009).

26. Li, H. *et al.* The Sequence Alignment/Map format and SAMtools. *Bioinforma. Oxf. Engl.* **25,** 2078–2079 (2009).

27. Ronquist, F. & Huelsenbeck, J. P. MrBayes 3: Bayesian phylogenetic inference under mixed models. *Bioinforma. Oxf. Engl.* **19,** 1572–1574 (2003).

28. Abella, J. *et al.* Kretzoiarctos gen. nov., the Oldest Member of the Giant Panda Clade. *PLoS ONE* **7,** e48985 (2012).

29. Wu, J. *et al.* Phylogeographic and Demographic Analysis of the Asian Black Bear (*Ursus thibetanus*) Based on Mitochondrial DNA. *PloS One* **10,** e0136398 (2015).

30. Yang, Z. PAML 4: phylogenetic analysis by maximum likelihood. *Mol. Biol. Evol.* **24,** 1586–1591 (2007).

31. Tedford, R. H. & Martin, J. Plionarctos, a Tremarctine Bear (Ursidae: Carnivora) from Western North America. *J. Vertebr. Paleontol.* **21,** 311–321 (2001).

32. Gustafson, P. The vertebrate faunas of the Pliocene Ringold Formation, south-central Washington. University of of Oregon, Mus. *Nat Hist Bull* **23,** 1–62 (1978).

33. Cahill, J. A. *et al.* Genomic evidence of geographically widespread effect of gene flow from polar bears into brown bears. *Mol. Ecol.* **24,** 1205–1217 (2015).

34. Orlando, L. *et al.* Recalibrating Equus evolution using the genome sequence of an early Middle Pleistocene horse. *Nature* **499,** 74–78 (2013).

35. Akaike, H. A new look at the statistical model identification. *IEEE Trans. Autom. Control* **19,** 716–723 (1974).

36. Onorato, D. P., Hellgren, E. C., van Den Bussche, R. A. & Doan-Crider, D. L. Phylogeographic Patterns within a Metapopulation of Black Bears (*Ursus americanus*) in the American Southwest. *J. Mammal.* **85,** 140–147 (2004).

37. Nachman, M. W. & Crowell, S. L. Estimate of the Mutation Rate per Nucleotide in Humans. *Genetics* **156,** 297–304 (2000).

38. Li, H. & Durbin, R. Inference of human population history from individual whole-genome sequences. *Nature* **475,** 493–496 (2011).

39. Wall, J. D. Estimating ancestral population sizes and divergence times. *Genetics* **163,** 395–404 (2003).

40. Wong, A. K. *et al.* A Comprehensive Linkage Map of the Dog Genome. *Genetics* **184,** 595–605 (2010).

41. Bidon, T., Schreck, N., Hailer, F., Nilsson, M. & Janke, A. Genome-wide search identifies 1.9 megabases from the polar bear Y chromosome for evolutionary analyses. *Genome Biol. Evol.* 7, 2010-2022 (2015).
